# Five Draft Genome Sequences of Historical Yersinia pestis Strains of Phylogroups 2.MED4 and 2.MED1 of the Medieval Biovar

**DOI:** 10.1128/mra.00044-22

**Published:** 2022-04-12

**Authors:** Alina N. Balykova, Lyubov M. Kukleva, Ekaterina A. Naryshkina, Galina A. Eroshenko, Vladimir V. Kutyrev

**Affiliations:** a Russian Research Anti-Plague Institute “Microbe”, Federal Service for Surveillance in the Sphere of Consumer Rights Protection and Human Welfare, Saratov, Russian Federation; University of Maryland School of Medicine

## Abstract

We announce the genome sequences of five historical highly virulent Yersinia pestis strains of the phylogroups 2.MED4 and 2.MED1 of the medieval biovar. They were the etiological agents of plague outbreaks with high mortality rates in the Northern Caspian Sea region at the end of the 19th century and beginning of the 20th.

## ANNOUNCEMENT

The plague pathogen, the highly virulent Gram-negative bacterium Yersinia pestis, is one of the most striking examples of a pathogen with high epidemic potential. The main subspecies, which is virulent for humans, includes the following biovars: antiqua, medieval, oriental, and possibly also intermedium (1). Our molecular investigations showed that strains of the phylogenetic branch 2.MED of the medieval biovar occupy about 93.3% of the territory of the natural foci in the Commonwealth of Independent States (1, 2). At the end of the 19th century and beginning of the 20th, numerous outbreaks of plague were registered in the Northern Caspian Sea region in Russia and Kazakhstan. Our studies prove that since 1912, outbreaks in the Northern Caspian Sea region have been caused by Y. pestis strains of the phylogenetic branches 2.MED1 and 2.MED4 of the medieval biovar (3, 4). The study of the genomes of the etiological agents of plague outbreaks in the Northern Caspian Sea region at the beginning of the 20th century is important for elucidating the causes of the high mortality rates among the population, as well as for historical reconstruction of the evolution of the medieval biovar of the plague pathogen.

The five strains of Y. pestis used in this study were isolated from natural foci in the Northern Caspian Sea region between 1925 and 1932 (Table 1). The strains were grown in LB medium (pH 7.2) for 24 to 48 h at 28°C; for DNA isolation, daily cultures of the strains were grown in LB liquid for 24 h at 28°C. DNA was extracted using the PureLink genomic DNA minikit (Invitrogen, USA). Genome sequencing was performed using the Ion Torrent PGM platform (Thermo Fisher Scientific, USA), according to the manufacturer’s instructions. DNA libraries were prepared using the Ion PGM reagent 400 kit and Ion 318 chip kit (Thermo Fisher Scientific). For each genome, raw short-read sequences were filtered, quality controlled, and *de novo* assembled using the Ion Torrent Suite v5.12.3 software package (https://github.com/iontorrent/TS) and Newbler v2.6 (5). Default parameters were used for all software. Finally, we obtained from 203 to 260 contigs for each genome (Table 1). The average genome size was 4.5 Mb, with an average peak depth of the obtained genomes of 53× and GC contents ranging from 47.46% to 47.54%. Plasmid sequences were identified using the software package DNASTAR Lasergene v15.3 (6). All strains had three plasmids (pMT, pCD, and pPCP), except Y. pestis 106, which had no pPCP. The final assemblies were annotated using the NCBI Prokaryotic Genome Annotation Pipeline (PGAP) v5.3 (7). Each genome contains 3,711 to 4,026 coding sequences.

**TABLE 1:** Accession numbers and basic statistics of the genome assemblies of Y. pestis strains

Strain	Isolation source, yr	SRA accession no.	GenBank assembly accession no.	Data production metrics		Total length (bp)	No. of contigs	No. of contigs >1 kb	Peak depth (×)	*N*_50_ (bp)	Total no. of genes	No. of coding genes	%GC
				No. of raw reads	Length of raw reads (bp)								
36	Spermophilus pygmaeus, 1925	SRR17333920	JAJTSR000000000	1,169,251	283,896,647	4,518,558	260	213	58	36,008	4,170	3,747	47.54
70	Fleas, 1926	SRR17333916	JAJTSV000000000	840,127	223,784,416	4,516,182	244	196	50	37,521	4,168	3,711	47.46
106	Meriones meridianus, 1928	SRR17333918	JAJTST000000000	1,580,426	405,381,148	4,488,175	220	174	83	45,341	4,119	3,803	47.48
174	Human, 1932	SRR17333917	JAJTSU000000000	1,106,572	283,611,903	4,680,401	251	182	53	48,683	4,315	4,026	47.54
107	Human, 1929	SRR17333919	JAJTSS000000000	853,040	215,132,355	4,585,677	203	170	38	47,826	4,188	3,785	47.53

The obtained nucleotide sequences of the genomes of historical strains of Y. pestis are pivotal for understanding the patterns of microevolution and the spatiotemporal distribution of the medieval biovar in the Caspian Sea region.

### Data availability.

The whole-genome shotgun projects and raw sequencing reads have been deposited at NCBI GenBank under BioProject accession number PRJNA792459 and are available under the accession numbers listed in Table 1.
